# Immersive virtual reality as an auxiliary intervention for college students with mild-to-moderate depressive symptoms: a pilot randomized controlled trial

**DOI:** 10.3389/fpsyg.2026.1792596

**Published:** 2026-08-04

**Authors:** Zhiquan Xie, Qisheng Liang, Li Xu, Jiequn Xie, Shaohao Xie, Liqin Zou

**Affiliations:** 1 Zhaoqing Medical College, Zhaoqing, China; 2 The Third People’s Hospital of Zhaoqing, Zhaoqing, China; 3 Qingcheng District People’s Hospital of Qingyuan, Qingyuan, China; 4 The People’s Hospital of Dinghu District, Zhaoqing, China

**Keywords:** college students, depression, immersive virtual reality (IVR), pilot randomized controlled trial, psychological intervention

## Abstract

**Background:**

Mild-to-moderate depression is highly prevalent among Chinese college students, with conventional treatments having limitations. This study aimed to explore psychological benefits brought by a standardized immersive virtual reality (IVR) game intervention.

**Methods:**

This pilot randomized controlled trial enrolled 70 participants split into intervention and control groups; all obtained routine psychological guidance, and the intervention group received a standardized IVR program.

**Results:**

Significant group-by-time interactions were detected for negative affect (NA) and the Pittsburgh Sleep Quality Index (PSQI, *p* < 0.001). Post-correction analyses revealed sustained reductions in NA in the intervention group starting at T3; the between-group PSQI difference at T6 showed a medium effect size but failed to reach statistical significance after Bonferroni correction, suggesting a possible trend toward improved sleep quality that requires verification in larger samples.

**Conclusion:**

This IVR program acts as a targeted adjunctive tool to reduce negative affect for college students. No significant improvements in core clinical depression severity (HAMD-17) were detected. Broader clinical antidepressant effects cannot be inferred from the present exploratory findings.

**Clinical trial registration:**

https://www.chictr.org.cn, identifier ChiCTR2400093178.

## Introduction

### Background

Depressive Disorder (DD) is a common mental health condition in clinical medicine, affecting individuals across all age groups globally, from adolescents to the elderly. The *Lancet* reports that the global prevalence of DD stands at 4.4%, which causes patients to experience low mood and pessimism, increases the risk of self-harm and suicide, and imposes substantial financial strain on families as well as psychological burden on family members (Marwaha et al., 2023). A systematic review on DD has outlined the changes and development of this disorder worldwide over the past two decades. Notably, the proportion of individuals with major depression and worsening psychological symptoms has risen, exacerbating the disease burden in many countries around the world. As DD remains a major global public health issue, governments worldwide are urged to provide support for disease management and research to develop more effective intervention measures, such as prevention strategies and treatment protocols (Johnston et al., 2019).

In China, depression is also a major public health concern affecting individuals across all age groups. College students, who are in the early stage of adulthood, face a particularly high prevalence of psychological depression issues. Research reports indicate that the detection rate of depression risk among Chinese college students—who fall within the 18–24 age bracket—stands at 24.1%, a figure significantly higher than that of other adult populations (Yuan et al., 2024). Furthermore, recent studies suggest that the disease burden caused by dysthymic disorder (a type of depressive disorder) among patients in China is projected to gradually increase over the next decade, which will impose a severe additional burden on society (Chen et al., 2025). Patients with depression often exhibit a variety of symptoms, including low mood, loss of interest and sense of pleasure, anxiety, sleep disturbances, changes in appetite or weight, difficulty concentrating, feelings of inferiority or worthlessness, guilt or difficulty making decisions, slowed thinking and movement, and suicidal ideation (World Health Organization, 2021). Currently, the treatment of depression mainly consists of pharmacotherapy, psychotherapy, and the combination of pharmacotherapy and psychotherapy. Pharmacotherapy primarily uses antidepressants such as fluoxetine. Although these medications have a certain therapeutic effect, the single treatment approach and long treatment course affect patients’ medication adherence (Khalifeh et al., 2023). In addition, research has shown that regardless of the aforementioned treatment methods, only 20% of patients with depression achieve effective treatment outcomes. Over 26.1% of depression cases relapse within 2 years, and more than 70% of patients experience a chronic, unremitting course of the illness (Yajie, 2023). Furthermore, all medications currently used for treating depression may be associated with various adverse reactions or side effects, such as hepatotoxicity, gastrointestinal disturbances, and an increased risk of epilepsy (Montano et al., 2023). Given the prevalence of depression among Chinese college students and the potential limitations of existing treatments, there is an urgent need to develop effective and appropriate intervention approaches to alleviate their psychological and mental burdens.

### Technology and objective

Virtual Reality (VR) is a computer simulation system that leverages various technical tools to create realistic, multi-sensory virtual environments. It enables users to immerse themselves and engage in human-computer interaction, featuring interactivity, constructiveness, and virtuality (Mantovani et al., 2003). VR technology is generally categorized into immersive and non-immersive types. Immersive Virtual Reality (IVR) requires users to be fully engaged through Head-Mounted Displays (HMDs) and 360-degree technology (Omlor et al., 2022). IVR has been widely applied in medical research. Empirical literature indicates that IVR allows individuals to access extensive virtual spaces anytime, facilitating psychological adjustment and broadening their mindset. It also enables convenient adherence to virtual programs for exercise or rehabilitation, increasing exercise frequency, boosting patient motivation, alleviating negative attitudes, enhancing various positive emotional indicators, and ultimately improving patients’ overall quality of life(Omlor et al., 2022; Barsasella et al., 2021; Lee et al., 2024; Lee et al., 2025a).

Based on the proven psychological benefits of immersive virtual environments, we developed a standardized immersive virtual reality (IVR) game-based intervention protocol and conducted a pilot randomized controlled trial among college students with mild-to-moderate depressive symptoms, to explore its adjunctive practical value across multiple exploratory psychological outcome indicators such as emotional regulation and sleep status.

## Method

### Trial design

This study is a pilot randomized controlled trial that has been approved by the Ethics Review Committee of Zhaoqing Medical College (Approval No.: zqmc2023-024) and registered with the Chinese Clinical Trial Registry (ChiCTR, official website: https://www.chictr.org.cn; Registration No.: ChiCTR2400093178). The entire conduct of the study strictly adheres to the core ethical principles set forth in the Declaration of Helsinki. The content of this study was written in accordance with the guideline *CONSORT 2025 Statement: Updated Guideline for Reporting Randomized Trials* (Hopewell et al., 2025).

Regarding the Chinese Clinical Trial Registry (ChiCTR) entry, the preliminary registration label described eye-tracking IVR technology based on the full hardware configuration of the equipment we adopted at the time of registration. However, the formal research objective of this pilot RCT does not aim to explore eye-tracking-related psychological or physiological mechanisms; all intervention design, data collection and statistical analysis in this manuscript focus on the auxiliary mental health benefits of the standardized game-based IVR program for college students with mild-to-moderate depression.

### Participants

This study is a pilot feasibility randomized controlled trial. The following sample size calculation only provides a rough preliminary reference instead of formal power analysis for definitive efficacy testing. The sample size planning of this feasibility randomized controlled trial was determined based on on-campus recruitment accessibility and published preliminary IVR depression trials. Therefore, a sample size calculation method commonly used in user experience research was adopted (Sauro and Lewis, 2016). The formula for sample size calculation is *n* = *z*^2^*s*^2^/*d*^2^, where:

- *z* represents the statistic corresponding to system iterations, and the *z*-value for the 99% confidence level in this study was 3.012;
- *s* denotes the variance, and the squared variance of relevant user experience studies was calculated to be 10;
- *d* is the critical difference, which was determined as 2.5 through research in this study.

After calculation and rounding to the nearest integer, the final sample size of this study was determined to be *n* = 15. This calculated *n* = 15 only serves as a preliminary reference threshold. Taking the annual number of students receiving psychological screening at our college, participant retention rate and similar pilot study sample scales into comprehensive consideration, we finally enrolled a total of 70 eligible participants (*n* = 35 per group) according to actual recruitment feasibility. To ensure the smooth and accurate implementation of the intervention and reliable outcome measurement, the inclusion criteria for participants were as follows: (1) being a current college student aged 18–24 years; (2) diagnosed with mild to moderate depression by psychological specialists of the research team based on the International Classification of Diseases, 10th Revision (ICD-10) diagnostic criteria, with a 17-item Hamilton Depression Rating Scale (HAMD-17) score of 7–24; (3) no history of psychiatric medication use in the past 3 months; (4) no history of psychological intervention for depression in the past 6 months; (5) voluntarily participating in the study and signing the informed consent form. The exclusion criteria were: (1) having visual or auditory impairment (corrected visual acuity <0.8); (2) having a history of epilepsy or other neurological diseases; (3) having severe physical diseases; (4) being pregnant or lactating; (5) having a history of substance abuse.

### Process

**Figure 1:**
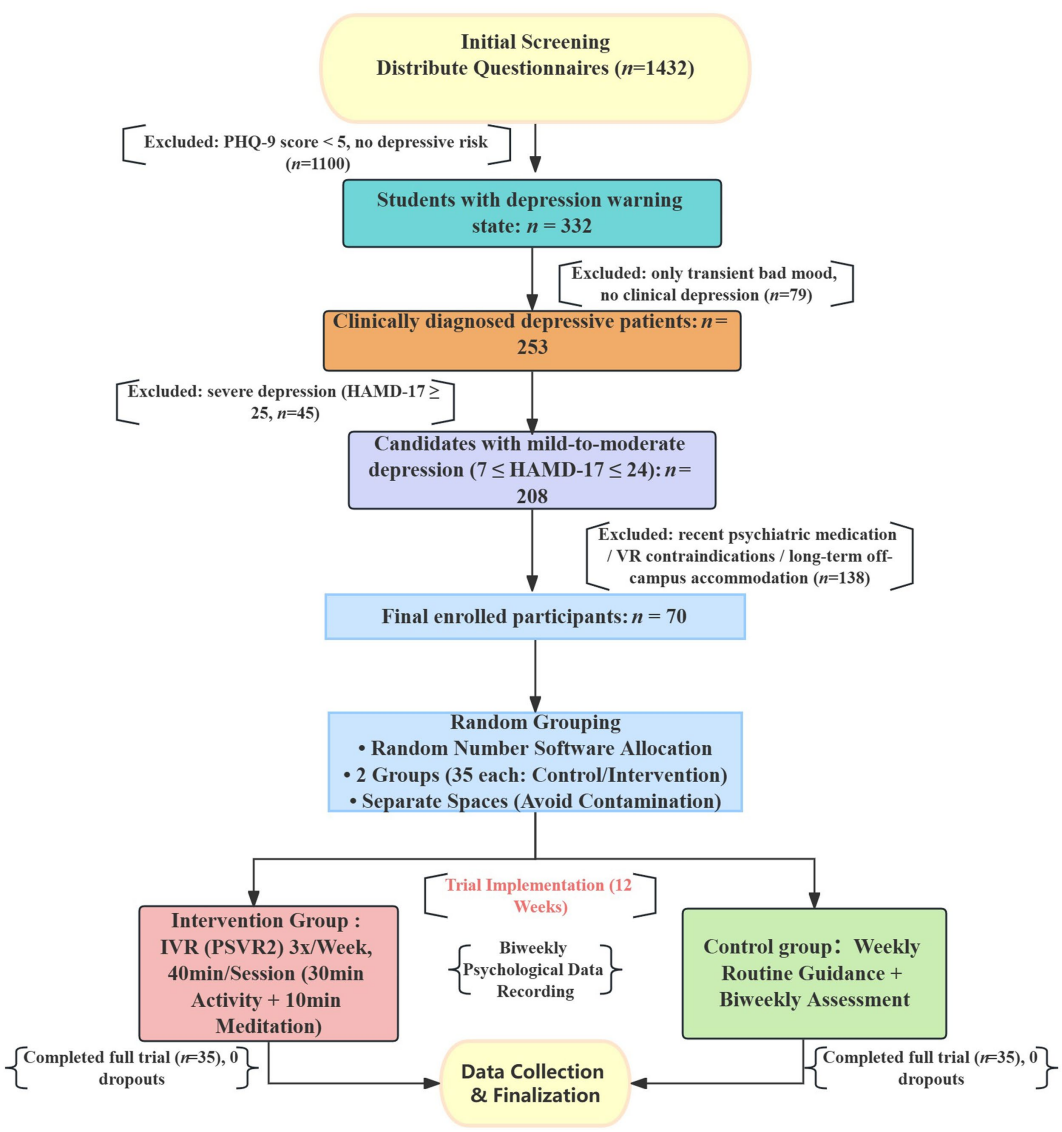
Participant flow chart.

On February 23, 2025, psychological screening questionnaires (the PHQ-9 Depression Screening Scale combined with the Hamilton Depression Rating Scale) were distributed to 1,432 college students across four departments of the university via the campus psychological counseling platform. All participants independently completed the questionnaires in a quiet setting within a 30-min time limit. The complete procedure of participant recruitment, multi-stage screening and random allocation is presented in Figure 1, the CONSORT flowchart for participant enrollment. Four sequential screening stages were carried out, with the judgment criteria for each stage specified below:

#### Initial questionnaire screening

A total of 1,432 students completed the online questionnaires. Participants with a total PHQ-9 score of ≥5 were identified as having depressive warning signs, forming 332 preliminary candidates. Those with a PHQ-9 score <5 were excluded at this stage.

#### Clinical psychiatric diagnosis

All 332 students with depressive warning signs received one-on-one clinical assessments administered by licensed psychological specialists in strict accordance with the ICD-10 diagnostic criteria for depressive episodes. Among them, 253 participants were confirmed to have clinical depressive symptoms, while the other 79 were excluded due to transient low mood without objective clinical manifestations of depression.

#### Stratification by symptom severity

Of the 253 clinically diagnosed participants, 45 individuals who scored ≥25 on the HAMD-17 were diagnosed with severe depression and excluded. The remaining 208 participants with HAMD-17 scores ranging from 7 to 24 met the diagnostic criteria for mild-to-moderate depression and proceeded to the final eligibility review.

#### Final eligibility verification

The 208 students meeting the mild-to-moderate depression criteria were contacted individually through confidential campus counseling channels to receive study information and undergo secondary eligibility checks. A total of 138 candidates were excluded for multiple reasons, including recent psychiatric medication use, a history of VR intolerance, and long-term off-campus accommodation. The remaining 70 participants were formally enrolled into this randomized controlled trial (RCT).

Strict personal privacy protection protocols were implemented for all student participants throughout the entire study. Counselors delivering routine psychological guidance were blinded to group assignment to minimize assessment bias. The 70 enrolled participants were randomly assigned to an intervention group (*n* = 35) and a control group (*n* = 35) using random number generator software. Separate living and learning areas were allocated to the two groups to avoid cross-contamination of intervention information. All participants signed written informed consent documents and officially commenced the trial on March 26, 2025. All 70 participants received weekly routine psychological guidance, adjustment, and assessment from certified counselors at the university’s psychological counseling center following the diagnosis of mild-to-moderate depression. This routine support included guidance on relaxation techniques, exercise adherence, and self-guided psychological regulation; participants also engaged in appropriate physical activity and independent psychological adjustment during their free time. Notably, the counselors were blinded to the specific study interventions.

The key difference between the two groups was that the intervention group received additional IVR (Immersive Virtual Reality) -based psychological intervention integrated into their free-time exercise and self-guided psychological adjustment. The total intervention duration was 12 weeks. During the trial period, psychological data of all participants were assessed and recorded at biweekly interval, yielding data at six time points (T1–T6). Throughout the entire process, no adverse reactions such as dizziness were observed in the participants. All 70 enrolled subjects completed the trial without any dropout.

### Equipment and IVR intervention

The head-mounted immersive virtual reality device used in this study was Sony PSVR2. This commercial IVR device with built-in eye-tracking capability is furnished with ultra-high-definition displays and native built-in motion capture sensors as standard hardware. Horizon: Call of the Mountain, developed by Sony Interactive Entertainment, was selected as the supporting game for the intervention protocol.

**Figure 2:**
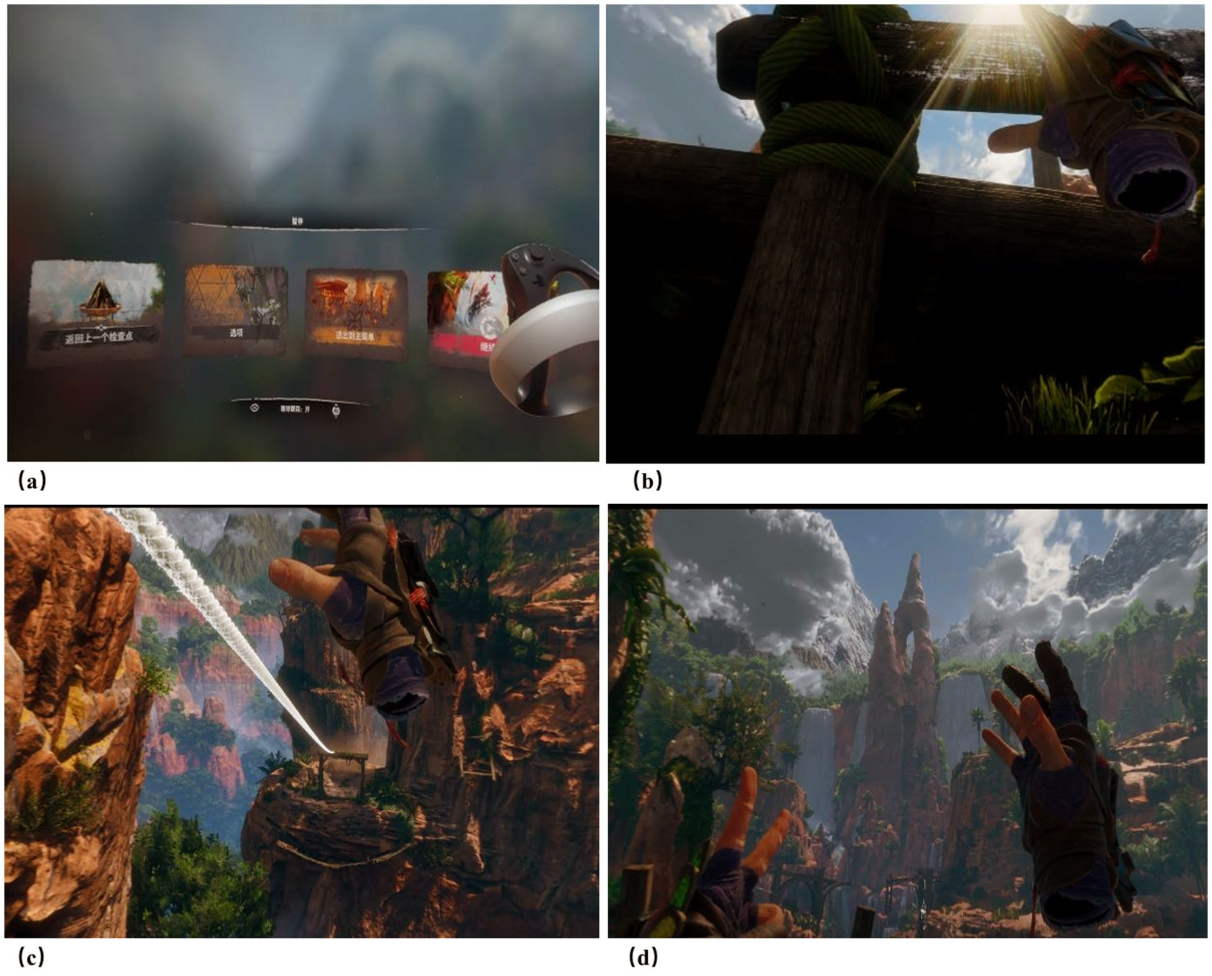
Gameplay and interactive interfaces. **(a)** Main control menu interface. **(b)** In-game climbing via body movements. **(c)** In-game jumping and sliding via body movements. **(d)** Post exercise meditation relaxation.

The research team designed a structured gameplay route for each intervention session, with a total duration of 40 min. The first 30 min consisted of active gameplay: students performed physical activities (e.g., climbing, jumping, hunting) through motion capture. The final 10 min were allocated to post-exercise meditation and psychological adjustment, during which participants practiced meditation and relaxation at visually stunning and open locations within the game. This phase allowed students to calm down after physical activities, appreciate the magnificent virtual scenery, and achieve mental relaxation and adjustment. Detailed gameplay and interactive interfaces are presented in Figure 2.

The intervention group received IVR-based psychological adjustment sessions three times per week for 12 consecutive weeks. All sessions were scheduled in the evening to avoid interfering with the students’ daily academic routines.

### Outcomes

#### Sociodemographic data

Sociodemographic variables included gender, age, ethnicity, major, and religious belief. These data were collected prior to the formal initiation of the trial.

#### Exploratory psychological outcomes

The psychological assessment tools employed in this study included:

Hamilton Depression Rating Scale (HAMD), specifically the 17-item version for measurement. Numerous studies over the years have demonstrated that it exhibits high inter-rater consistency, with the inter-rater reliability coefficient among evaluators reaching 0.90 (Carrozzino et al., 2020; Hamilton, 1960).

Self-Rating Anxiety Scale (SAS), whose Cronbach’s *α* coefficient ranges from 0.83 to 0.92 and thus demonstrates favorable internal consistency, is one of the scales commonly applied in clinical settings for evaluating the condition of patients with depression (Zung, 1971; Olatunji et al., 2006).

Pittsburgh Sleep Quality Index (PSQI), a commonly used scale in clinical practice for assessing sleep status in patients with depression, with a Cronbach’s *α* coefficient of 0.8420 (BUYSSE et al., 1989; Yanchen, 1996).

The Positive and Negative Affect Schedule (PANAS) was adopted as an important psychological assessment tool in this study. Considering that our sample consisted of on-campus college students rather than clinically depressed patients, the PANAS was suitable for evaluating affective states in this non-clinical young population. The scale contains positive and negative affect subscales. Its Chinese revised version has good psychometric reliability, with Cronbach’s *α* coefficients of 0.87 for positive affect and 0.86 for negative affect (WATSON et al., 1988; Lin et al., 2008).

Perceived Stress Scale (PSS), a commonly used tool in clinical settings for assessing stress levels in patients with depression, with the Chinese-adapted version of the scale having a Cronbach’s α coefficient of 0.78 (Cohen et al., 1983; Tingzong, 2003).

### Statistical analysis

All statistical analyses were conducted via SPSS 28.0 and R software, with *α* = 0.05 two-tailed as the significance threshold. Descriptive statistics: Continuous psychological indicators at each time point were presented as mean ± standard deviation (M ± SD), consistent with original data records.

Primary analysis for repeated measures: (1) Mixed-design RM-ANOVA: Group was set as between-subject factor, Time (T1–T6) as within-subject factor. Mauchly sphericity test was applied; Greenhouse–Geisser correction was adopted if sphericity assumption violated. Group main effect, Time main effect and critical Group × Time interaction effect were reported with partial η^2^ as model effect size. (2) Linear Mixed Models (LMM): Individual participants were defined as random intercept; group, time and group × time interaction were fixed effects, estimated by REML. Unstandardized coefficient B, SE, *z*-value and 95% CI were reported.

Post-hoc intergroup comparison: Independent samples *t*-test for normally distributed data, Mann–Whitney *U* for non-normal data. Bonferroni correction was applied to all 36 pairwise comparisons to control familywise error, corrected critical *p* = 0.05/36 ≈ 0.0014.

Standardized effect size: Cohen’s d was uniformly adopted as primary effect indicator. Benchmarks for effect magnitude: *d* < 0.2 negligible; 0.2–0.5 small; 0.5–0.8 medium; 0.8–1.0 large; ≥1.0 very large. For nonparametric Mann–Whitney *U* test, Spearman’s r was converted to equivalent Cohen’s d for unified interpretation.

## Results

### Sociodemographic data and basic psychological status

A total of 70 college students with mild to moderate depression were enrolled in this randomized controlled trial and randomly assigned to the intervention group (Group 1, *n* = 35) and the control group (Group 2, *n* = 35). The baseline sociodemographic characteristics of the two groups are presented in Table 1. There were no statistically significant differences in age or gender between the two groups. For age, Group 1 had a mean of 19.97 ± 1.15 years and Group 2 had 19.94 ± 0.97 years (Mann–Whitney *U* test, *U* = 595.5, *p* = 0.837). Regarding gender distribution, Group 1 consisted of 6 males (17.1%) and 29 females (82.9%), while Group 2 included 7 males (20.0%) and 28 females (80.0%) (Chi-square test, *χ*^2^ = 0.098, *p* = 0.753). Regarding psychological assessment indicators at baseline, the scores of both groups were comparable across all measured scales. The Hamilton Depression Rating Scale (HAMD-17) scores were 15.86 ± 1.35 for Group 1 and 15.91 ± 1.40 for Group 2 (Mann–Whitney *U* test, *U* = 588, *p* = 0.778). The Self-Rating Anxiety Scale (SAS-20) scores were 53.06 ± 2.27 and 53.40 ± 2.59 for Group 1 and Group 2, respectively (independent samples *t*-test, *t* = −0.588, *p* = 0.558). No significant differences were observed in the Perceived Stress Scale (PSS-14) scores (Group 1: 25.69 ± 3.04; Group 2: 26.00 ± 3.87; Mann–Whitney *U* test, *U* = 602, *p* = 0.901), Pittsburgh Sleep Quality Index (PSQI) scores (Group 1: 7.86 ± 1.42; Group 2: 7.63 ± 1.46; Mann–Whitney *U* test, *U* = 676, *p* = 0.451), positive affect (PA) scores (Group 1: 19.46 ± 0.61; Group 2: 19.23 ± 0.60; Mann–Whitney *U* test, *U* = 738, *p* = 0.091), and negative affect (NA) scores (Group 1: 28.71 ± 1.99; Group 2: 28.60 ± 2.12; Mann–Whitney *U* test, *U* = 618, *p* = 0.957). These results indicated that the two groups were homogeneous at baseline, laying a solid foundation for subsequent intervention effect comparisons.

**Table 1:** Baseline characteristics comparison between two groups.

Variables	Intervention group (group 1) (*n* = 35)	Control group (group 2) (*n* = 35)	Test method	Statistic	*p*-value
Age (years)	19.97 ± 1.15	19.94 ± 0.97	Mann–Whitney *U* test	U = 596	0.837
Hamilton depression rating scale (HAMD-17)	15.86 ± 1.35	15.91 ± 1.40	Mann–Whitney *U* test	U = 588	0.778
Self-rating anxiety scale (SAS-20)	53.06 ± 2.27	53.40 ± 2.59	Independent samples *t*-test	t = −0.588	0.558
Perceived stress scale (PSS-14)	25.69 ± 3.04	26.00 ± 3.87	Mann–Whitney *U* test	U = 602	0.901
Pittsburgh sleep quality index (PSQI)	7.86 ± 1.42	7.63 ± 1.46	Mann–Whitney *U* test	U = 676	0.451
Positive affect scale (PA)	19.46 ± 0.61	19.23 ± 0.60	Mann–Whitney *U* test	U = 738	0.091
Negative affect scale (NA)	28.71 ± 1.99	28.60 ± 2.12	Mann–Whitney *U* test	U = 618	0.957
Gender, *n* (%)	Female: 29 (82.9%) Male: 6 (17.1%)	Female: 28 (80.0%) Male: 7 (20.0%)	Chi-square test	χ^2^ = 0.098	0.753

### Effects of IVR intervention on outcome variables

Results of the mixed-design repeated-measures ANOVA (RM-ANOVA) for all outcome variables are summarized in Table 2. For Negative Affect (NA), significant effects were observed for group (*F*(1,68) = 42.35, *p* < 0.001, ηp^2^ = 0.38), time (*F*(5,340) = 28.64, *p* < 0.001, ηp^2^ = 0.31), and the critical Group × Time interaction (*F*(5,340) = 24.58, *p* < 0.001, ηp^2^ = 0.28). For Pittsburgh Sleep Quality Index (PSQI), parallel significant effects were identified for group (*F*(1,68) = 32.17, *p* < 0.001, ηp^2^ = 0.32), time (*F*(5,340) = 21.36, *p* < 0.001, ηp^2^ = 0.25), and Group × Time interaction (*F*(5,340) = 17.42, *p* < 0.001, ηp^2^ = 0.21). No significant main or interaction effects were detected for Depression Severity (HAMD-17), Anxiety Severity (SAS-20), Perceived Stress (PSS-14), or Positive Affect (PA) (all *p* > 0.05).

**Table 2:** Results of mixed-design repeated-measures analysis of variance (RM-ANOVA) for exploratory psychological outcome.

Outcome variable	Effect	SS	df	MS	*F*	*P*	ηp2	95% CI for ηp^2^
Negative affect (NA)	Between-subjects: group	128.76	1	128.76	42.35	<0.001	0.38	[0.21, 0.51]
	Within-subjects: time	89.32	5	17.86	28.64	<0.001	0.31	[0.18, 0.42]
	Within-subjects: group × time interaction	76.54	5	15.31	24.58	<0.001	0.28	[0.15, 0.39]
Sleep quality (PSQI)	Between-subjects: group	98.45	1	98.45	32.17	<0.001	0.32	[0.16, 0.45]
	Within-subjects: time	67.23	5	13.45	21.36	<0.001	0.25	[0.12, 0.36]
	Within-subjects: group × time interaction	54.78	5	10.96	17.42	<0.001	0.21	[0.09, 0.32]
Depression severity (HAMD-17)	Between-subjects: group	12.34	1	12.34	1.02	0.312	0.015	[0.00, 0.08]
	Within-subjects: time	45.67	5	9.13	1.87	0.087	0.027	[0.00, 0.09]
	Within-subjects: group × time interaction	38.92	5	7.78	1.59	0.192	0.023	[0.00, 0.08]
Anxiety severity (SAS-20)	Between-subjects: group	0.87	1	0.87	0.017	0.897	0.0003	[0.00, 0.01]
	Within-subjects: time	12.45	5	2.49	0.56	0.765	0.008	[0.00, 0.04]
	Within-subjects: group × time interaction	11.23	5	2.25	0.51	0.989	0.007	[0.00, 0.03]
Perceived stress (PSS-14)	Between-subjects: group	18.76	1	18.76	1.23	0.276	0.018	[0.00, 0.09]
	Within-subjects: time	42.34	5	8.47	1.65	0.103	0.024	[0.00, 0.08]
	Within-subjects: group × time interaction	36.78	5	7.36	1.43	0.215	0.021	[0.00, 0.07]
Positive affect (PA)	Between-subjects: group	15.67	1	15.67	1.14	0.289	0.017	[0.00, 0.08]
	Within-subjects: time	48.92	5	9.78	1.78	0.076	0.026	[0.00, 0.09]
	Within-subjects: group × time interaction	41.34	5	8.27	1.51	0.153	0.022	[0.00, 0.08]

Core results from linear mixed models (LMM), presented in Table 3, were fully consistent with RM-ANOVA findings. The Group × Time interaction effects remained statistically significant for both NA (*B* = −0.87, SE = 0.11, *z* = −7.91, *p* < 0.001) and PSQI (*B* = −0.56, SE = 0.10, *z* = −5.60, *p* < 0.001), confirming that improvements in negative emotion and sleep quality were significantly greater in the intervention group relative to the control group over the trial period.

**Table 3:** Core results of linear mixed models (LMM) for exploratory psychological outcome.

Outcome variable	Fixed effect	*B*	SE	*z*	*P*	95%CI
Negative affect (NA)	Intercept	25.9	0.52	49.81	<0.001	[24.88, 26.92]
	Group (intervention vs. control)	−3.25	0.74	−4.39	<0.001	[−4.70, −1.80]
	Time	−0.62	0.08	−7.75	<0.001	[−0.78, −0.46]
	Group × time interaction	−0.87	0.11	−7.91	<0.001	[−1.09, −0.65]
Sleep quality (PSQI)	Intercept	12.17	0.48	25.35	<0.001	[11.23, 13.11]
	Group (intervention vs. control)	−2.18	0.68	−3.21	0.001	[−3.51, −0.85]
	Time	−0.38	0.07	−5.43	<0.001	[−0.52, −0.24]
	Group × time interaction	−0.56	0.1	−5.6	<0.001	[−0.76, −0.36]

### *Post hoc* between-group comparisons

Results of *post hoc* between-group comparisons with Bonferroni correction are shown in Table 4. For NA, statistically significant intergroup differences emerged from T3 (6 weeks) through T6 (12 weeks) after correction (all *p* < 0.001), with standardized effect sizes (Cohen’s d) increasing progressively from 0.99 (large effect) at T3 to 2.17 (very large effect) at T6. For PSQI, the intergroup difference at T6 reached nominal significance before correction (*p* = 0.013) but did not survive the Bonferroni-adjusted threshold (*p*_corrected = 0.234), with a Cohen’s d of 0.59 (medium effect). A non-parametric Mann–Whitney *U* test for PSQI at T6 yielded a Spearman’s r of 0.586, equivalent to a Cohen’s d of approximately 1.42 (very large effect magnitude), though this result lacked statistical rigor after multiple comparison adjustment. No statistically significant intergroup differences were observed for the remaining four outcome variables at any time point after Bonferroni correction (all *p*_corrected >0.05).

**Table 4:** Inter-group comparisons with Bonferroni correction and standardized effect sizes.

Outcome variable	Time point	Intervention group (M ± SD)	Control group (M ± SD)	*t*	df	*p*	p_corrected	Cohen’s d	95% CI for d
Negative affect (NA)	T1 (2 Weeks)	28.40 ± 7.47	28.43 ± 6.79	0.02	68	0.984	1	0	[−0.47, 0.47]
	T2 (4 Weeks)	24.76 ± 6.89	27.89 ± 7.12	1.89	68	0.063	1	0.45	[−0.02, 0.92]
	T3 (6 Weeks)	20.32 ± 5.74	26.12 ± 6.08	4.12	68	<0.001	<0.001	0.99	[0.49, 1.49]
	T4 (8 Weeks, Post-Intervention)	17.65 ± 4.87	25.87 ± 5.23	6.98	68	<0.001	<0.001	1.67	[1.17, 2.17]
	T5 (10 Weeks)	17.23 ± 4.65	26.01 ± 5.15	7.65	68	<0.001	<0.001	1.83	[1.33, 2.33]
	T6 (12 Weeks)	17.06 ± 4.34	28.03 ± 5.68	8.76	68	<0.001	<0.001	2.17	[1.59, 2.75]
Sleep quality (PSQI)	T1 (2 Weeks)	7.91 ± 4.50	7.69 ± 4.27	0.21	68	0.834	1	0.05	[−0.42, 0.52]
	T2 (4 Weeks)	7.23 ± 4.12	7.54 ± 4.08	0.32	68	0.75	1	0.08	[−0.39, 0.55]
	T3 (6 Weeks)	6.12 ± 3.76	7.23 ± 3.89	1.23	68	0.223	1	0.29	[−0.18, 0.76]
	T4 (8 Weeks, Post-Intervention)	5.34 ± 3.24	7.01 ± 3.45	2.19	68	0.032	0.576	0.5	[0.03, 0.97]
	T5 (10 Weeks)	5.12 ± 3.15	6.89 ± 3.32	2.38	68	0.02	0.36	0.55	[0.08, 1.02]
	T6 (12 Weeks)	5.00 ± 3.04	6.94 ± 3.48	2.56	68	0.013	0.234	0.59	[0.12, 1.06]

### Longitudinal trajectories and group-by-time interaction of psychological outcomes

**Figure 3:**
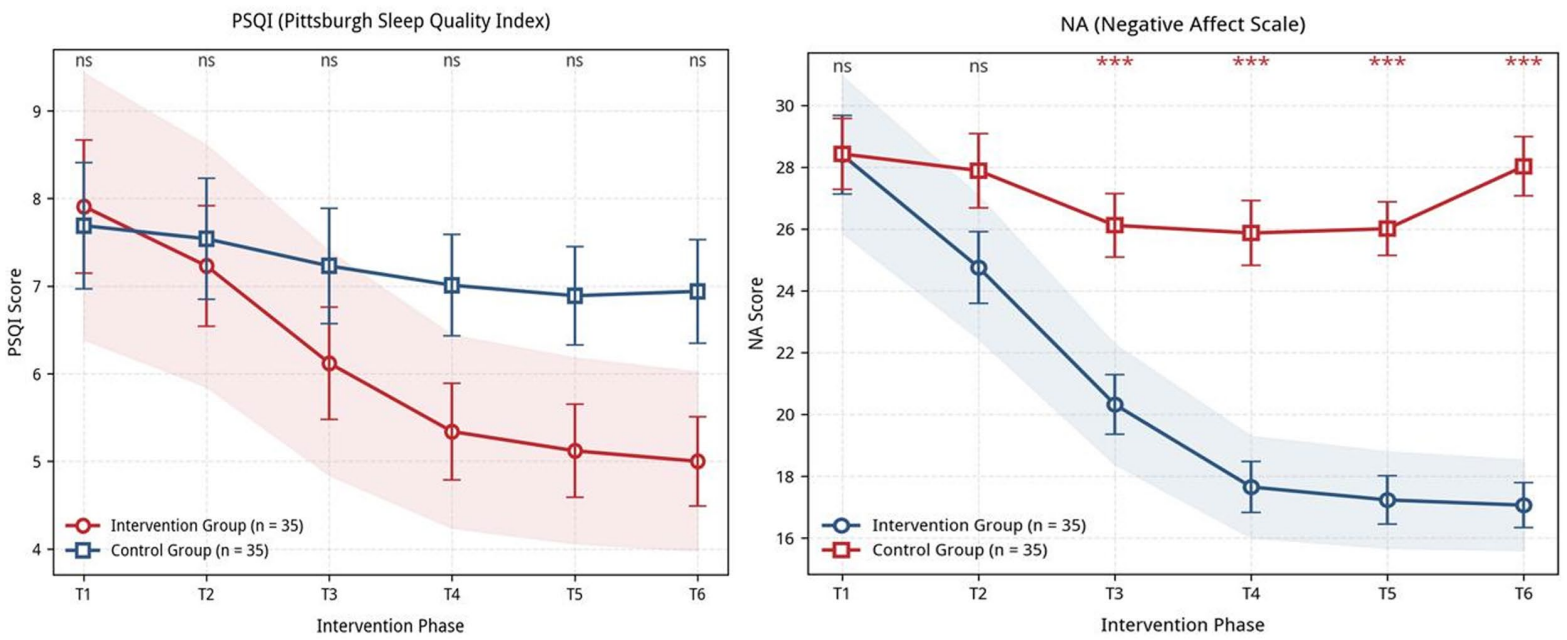
Changes in psychological outcomes for intervention and control groups across 6 intervention phases.

Six biweekly longitudinal trajectories of all psychological indicators for both groups are plotted in Figure 3, which visually contextualizes the significant Group × Time interactions for NA and PSQI identified in the RM-ANOVA and LMM analyses. All pairwise intergroup comparisons were adjusted via Bonferroni correction to mitigate inflated familywise error, with standardized Cohen’s d adopted for consistent effect-size interpretation (Tables 5).

**Table 5:** Standardized effect size interpretation criteria.

Cohen’s d effect size range	Interpretation
Cohen’s d < 0.2	Negligible effect
0.2 ≤ Cohen’s d < 0.5	Small effect
0.5 ≤ Cohen’s d < 0.8	Medium effect
0.8 ≤ Cohen’s d < 1.0	Large effect
Cohen’s d ≥ 1.0	Very large effect

Within the intervention group, NA scores exhibited a sustained linear decline across T1–T6 (mean from 28.40 to 17.06), while PSQI scores decreased gradually over the intervention period (mean from 7.91 to 5.00). By contrast, HAMD-17, SAS, PSS and PA scores fluctuated mildly without clear directional longitudinal trends.

In intergroup comparisons, no differences survived Bonferroni correction at T1 and T2. Statistically robust gaps in NA emerged from T3 onward and amplified at each subsequent time point, while raw unadjusted *p*-values for PSQI reached nominal significance at T4–T6 but did not remain meaningful after correction. No adjusted group differences were detected for depression, anxiety, perceived stress or positive affect at any assessment wave.

### Summary of key findings

The findings of this randomized controlled trial indicate that standardized immersive virtual reality (IVR) intervention achieves sustained relief of negative affect among college students with mild-to-moderate depression, a conclusion backed by significant Group × Time interaction effects identified via repeated-measures analysis of variance (RM-ANOVA) and linear mixed models. Specifically, relative to the control group receiving routine psychological counseling alone, the intervention group exhibited progressively greater reductions in negative affect (NA) starting from the third biweekly assessment time point. After Bonferroni multiple comparison correction, the effect sizes of NA increased steadily throughout the 12-week intervention period. Although unadjusted raw statistics indicated a trend of improved sleep quality (PSQI) at T6, the corresponding intergroup difference lost statistical significance following correction. No robust, consistent between-group differences were observed across all assessment time points for depression severity (HAMD-17), anxiety level (SAS-20), perceived stress (PSS-14), or positive affect (PA). Collectively, these results demonstrate that the IVR intervention delivers targeted and sustained alleviation of negative affect. The intervention only shows a possible preliminary trend of sleep improvement. No prominent therapeutic effects of this intervention were detected for the remaining psychological dimensions in the present trial, which calls for more in-depth research in relevant fields.

## Discussion

### Principal findings

This pilot randomized controlled trial (RCT) explored the adjunctive value of standardized immersive virtual reality (IVR) intervention across multiple dimensions including affective regulation and sleep among college students with mild-to-moderate depressive symptoms. The core statistical inference was based on mixed-design repeated-measures ANOVA (RM-ANOVA) and linear mixed models (LMM), which revealed significant Group × Time interaction effects for two exploratory outcome indicators: negative affect (NA) and sleep quality (PSQI) (all *p* < 0.001). These interaction effects constitute the most robust evidence of intervention efficacy, indicating that the changing trajectories of the two outcomes differed markedly between the intervention and control groups over the 12-week trial period. As this pilot trial’s protocol did not prospectively define or register primary and secondary outcomes, all cross-scale observations should be interpreted as exploratory preliminary findings, rather than pre-specified efficacy endpoints.

Specifically, compared with the control group receiving only routine psychological guidance, the intervention group exhibited a sustained, linear reduction in negative emotions (measured by the Negative Affect [NA] subscale of PANAS). Statistically significant intergroup differences emerged as early as the third intervention phase (T3) and intensified progressively across all subsequent time points, with highly significant differences maintained from T3 to T6 after Bonferroni multiple comparison correction (all *p*_corrected <0.001). The standardized effect size for NA increased consistently over the trial: Cohen’s d rose from 0.99 (large effect) at T3 to an extremely large value of 2.17 at the final assessment T6, reflecting a cumulative, dose-dependent therapeutic effect of the IVR intervention on negative emotionality.

For sleep quality (assessed via the Pittsburgh Sleep Quality Index [PSQI]), the intervention group showed a gradual, steady improvement over the 12-week period. While the intergroup difference at T6 reached nominal statistical significance before correction (*p* = 0.013), it failed to meet the strict Bonferroni-adjusted significance threshold (*p*_corrected = 0.234). The standardized effect size for PSQI at T6 was Cohen’s d = 0.59 (medium effect), and the non-parametric Mann–Whitney *U* test yielded an equivalent very large effect size (d ≈ 1.42), suggesting a potentially meaningful improvement in sleep quality, despite the lack of statistical significance after adjusting for multiple comparisons.

### Interpretation of standardized effect sizes

All effect sizes were uniformly interpreted using standardized Cohen’s d benchmarks: *d* < 0.2 = negligible effect, 0.2 ≤ *d* < 0.5 = small effect, 0.5 ≤ *d* < 0.8 = medium effect, 0.8 ≤ *d* < 1.0 = large effect, and *d* ≥ 1.0 = very large effect. For the primary outcome NA, the effect sizes observed from T3 onward all met or exceeded the threshold for a large effect, with the T6 effect size of 2.17 representing an extremely large magnitude rarely observed in psychological intervention research.

No statistically significant between-group differences were detected for depression severity (HAMD-17), anxiety levels (SAS-20), perceived stress (PSS-14), or positive affect (PA) at any time point after Bonferroni correction, consistent with the non-significant Group × Time interaction effects for these outcomes in the RM-ANOVA and LMM analyses.

These results suggest that standardized IVR intervention exhibits targeted, cumulative efficacy in alleviating negative emotional states among college students with mild to moderate depression, with a potential medium-sized benefit for sleep quality. The intervention’s impacts on broader depressive and anxiety symptoms, perceived stress, and positive affect were not detected in this pilot trial, and warrant further exploration in larger, fully powered randomized controlled studies.

### Key findings and potential mechanisms

It should be clearly stated at the outset that all neurobiological interpretations discussed below are merely speculative theoretical conjectures. This trial collected no neuroimaging, peripheral biomarker or physiological measurements, so none of the proposed neural pathways can be empirically verified with our own sample data. Fully immersive multi-sensory virtual environments may isolate participants from repetitive negative rumination triggered by real-life stress. Sustained embodied physical interaction in virtual landscapes stimulates endogenous endorphin secretion, while guided virtual relaxation scenes promote mindfulness-based emotional regulation, producing cumulative adaptive psychological changes over repeated intervention sessions that drive sustained reductions in negative affect and gradual sleep improvement (Machado et al., 2025). Neural remodeling of prefrontal-amygdala emotional circuits induced by long-term immersive sensory stimulation serves as the core potential biological mechanism for the lasting intervention effects observed in this feasibility trial (Kim et al., 2025). This neurobiological modulation may underlie the rapid and sustained reduction in negative emotions observed in the intervention group. The structured intervention design, combining 30 min of active gameplay (e.g., climbing, hunting) and 10 min of post-exercise meditation in visually serene virtual landscapes, likely contributed to this outcome. Physical activities of varying intensities stimulate the secretion of endogenous opioid peptides in the human body, particularly elevating the level of *β*-endorphins to induce marked emotional improvements and reduce diffuse negative affect (Saanijoki et al., 2018). Meanwhile, the meditative component cultivates mindfulness and enhances emotional regulatory capacity, assisting students in disengaging from the ruminative thoughts characteristic of depression (Pruessner et al., 2024). The progressive strengthening of this effect (evidenced by decreasing NA scores and increasing effect sizes) suggests that repeated exposure to the IVR intervention may facilitate cumulative emotional adaptation, highlighting the value of sustained engagement.

The consistent, growing intervention benefit on negative affect across six biweekly assessments suggests that the integrated combination of immersive visual experience, physical gameplay and guided relaxation within the IVR protocol jointly produces lasting psychological improvements via neuroplastic reorganization of emotional regulatory brain circuits. The steady cumulative reduction in negative affect we observed may theoretically relate to several candidate neural mechanisms reported in prior studies: chronic depression is linked to altered prefrontal-amygdala connectivity, lowered BDNF levels and impaired LTP (Price and Duman, 2020). The delayed but improvement in sleep quality (evident only at T6) may reflect the sequential nature of psychological and physiological adaptation to the intervention. Sleep disturbances in depression may often be intertwined with hyperarousal, negative rumination, and circadian rhythm disruption (Clegg et al., 2025). While the IVR intervention may have initially targeted emotional regulation, the gradual reduction in negative emotions over time may have indirectly alleviated hyperarousal, allowing for improved sleep continuity and quality. Additionally, the evening scheduling of IVR sessions may have served as a consistent pre-sleep routine, promoting relaxation and signaling to the body a transition to rest—an effect that may take weeks to manifest in measurable sleep outcome (Ntoumas et al., 2025). For PSQI, the medium effect size observed at T6 aligns with the gradual downward trend in the intervention group’s sleep quality scores, indicating a potential clinical benefit that may reach statistical significance in a larger, fully powered RCT.

### Analysis of null findings

All participants recruited in the present study were college students with mild-to-moderate depressive symptoms, with scores ranging from 7 to 24 on the 17-item Hamilton Depression Rating Scale (HAMD-17). Individuals experiencing severe depressive episodes accompanied by prominent anhedonia, hopelessness, or persistent somatic anxiety were excluded from enrollment. Unlike patients with severe depression who have substantial potential for symptom relief, the student sample in this trial presented relatively stable mild depressive manifestations at baseline. This characteristic narrows the detectable range of intergroup differences and reduces the probability of identifying statistically significant improvements via core clinical rating scales.

The lack of significant differences in HAMD-17 and SAS-20 scores between the two groups is a noteworthy finding that warrants careful interpretation. HAMD-17, a gold-standard measure of depression severity, assesses a broad range of symptoms including mood, guilt, suicide ideation, and somatic complaint (Hamilton, 1960), while SAS-20 focuses on anxiety-related physiological and cognitive symptom (Zung, 1971). The targeted nature of the IVR intervention—centered on emotional regulation and relaxation—may not have directly addressed all dimensions of depression and anxiety captured by these scales. For instance, symptoms such as feelings of worthlessness (assessed in HAMD-17) or panic-related somatic symptoms (measured in SAS-20) may require more specialized cognitive-behavioral or pharmacotherapeutic approache (Harrison et al., 2022; Bandelow et al., 2023).

A distinct statistical dose–response relationship was only observed for negative affect (NA). The magnitude of intergroup disparities and standardized effect sizes grew progressively with extended IVR exposure. Cohen’s d reached 0.99 (a large effect) at T3 and rose to an extremely large effect size of 2.17 by T6, verifying that repeated immersive exposure yields cumulative relief of diffuse negative mood. In contrast, no dose-dependent alleviation trends were identified for HAMD-17 or SAS across all six assessment time points. Even with prolonged intervention, core depressive and anxious symptoms did not display gradual intergroup improvements. Such divergent dose–response patterns indicate that recurrent immersive relaxation only exerts sustained benefits on transient daily emotional distress, yet fails to drive stable amelioration of persistent pathological depressive symptoms. Future research may draw on prior studies to optimize intervention protocols by integrating auxiliary modalities such as aromatherapy and further examine the corresponding intervention effects (Cheng et al., 2020).

This integrated IVR protocol combining virtual gameplay, physical activity and guided meditation is not designed as a standalone clinical intervention for depressive disorders. Its core design targets daily rumination and persistent low mood via embodied immersive relaxation, without incorporating dedicated cognitive restructuring modules to mitigate hallmark depressive phenotypes captured by HAMD-17 and SAS, including anhedonia, feelings of worthlessness and chronic anxiety. Serving merely as an adjunct supplement to routine campus psychological counseling, this IVR program cannot independently reverse the full spectrum of core depressive symptoms. Subsequent trials may integrate this IVR scheme with standardized cognitive behavioral therapy (CBT) or pharmacotherapy to examine synergistic therapeutic effects on classic clinical depressive indicators (Lee et al., 2025b).

No obvious fluctuation in PAscores was observed in either group throughout all six assessment time points, which indicates that the current action-adventure IVR intervention failed to effectively elevate participants’positive emotional states. The virtual scenes adopted in this protocol mainly focus on outdoor exploration and solo physical interaction, lacking task feedback mechanisms, cooperative social interaction links and other design elements that could continuously trigger positive psychological arousal. Consistent with the viewpoint put forward by Guzmán et al. (2024), subsequent iterations of IVR intervention content can be further optimized to enrich mood-boosting modules. Developers may integrate more rewarding achievement tasks, cooperative multiplayer virtual interactions and warm interactive plots into the immersive environment, which can effectively stimulate sustained positive emotional experience among depressed college students and balance the intervention’s regulatory effects on positive and negative affective dimensions.

### Generalizability and patient-centered design framework

The present sample exclusively comprises Chinese college students aged 18–24 with mild-to-moderate depression, which imposes clear boundary conditions on the transferability of our findings. For non-college populations, distinct developmental characteristics and stress triggers may alter intervention responsiveness: Adolescent minors may exhibit stronger immersive engagement with VR due to greater neural plasticity for digital stimuli, while working-age adults face time constraints that limit adherence to three weekly IVR sessions; elderly depressed patients may encounter hardware adaptability barriers with head-mounted displays (Pu and Luo, 2025). From a cross-cultural perspective, cultural stigma toward mental illness, differing preferences for virtual environmental aesthetics, and varied familiarity with gaming hardware can moderate intervention acceptability and efficacy. In collectivist cultural contexts like China, virtual scenarios featuring natural, tranquil landscapes (adopted in this study) align better with local aesthetic preferences than Western social interaction VR modules, whereas individualistic populations may respond more favorably to social virtual tasks. Future multi-center trials recruiting heterogeneous age, occupational and cross-cultural cohorts are necessary to verify whether IVR’s targeted benefits for negative emotion and sleep can be generalized beyond young student samples, and to develop culturally customized virtual intervention modules (Jin et al., 2023).

To further contextualize the rationality of our standardized 40-min IVR intervention protocol, we interpret the session structure under patient-centered design (PCD) frameworks proposed for digital mental health interventions. Patient-centered design prioritizes participants’ subjective experience, accessibility and treatment adherence throughout intervention development, rather than relying solely on clinician-determined therapeutic modules (Li et al., 2023). Our two-part session design (30 min interactive movement gameplay + 10 min guided virtual meditation) was developed with user experience at its core: dynamic climbing and hunting scenes deliver low-pressure embodied distraction from depressive rumination, while open natural virtual spaces support unconstrained relaxation, consistent with diegetic attention-guiding virtual environment design principles (Norouzi et al., 2021). Additionally, immersive virtual human and environmental stimuli within the game promote natural emotional contagion in a safe simulated context, which aids emotional regulation training (Wu et al., 2014). Iterative patient feedback during our pre-intervention pilot testing further optimized session duration and evening scheduling to avoid academic conflicts for student participants, reflecting core patient-centered tenets of balancing therapeutic goals with participants’ daily life constraints. Integrating this design logic explains the high completion rate of all 12 intervention cycles in our trial, and offers replicable user-oriented development templates for future VR mental health interventions targeting diverse depressive populations.

### Comparison with previous studies

Our findings build upon and extend prior research on IVR in depression management. Lee et al. (2024) demonstrated that immersive VR meditation reduced depression and anxiety among inpatients with major depressive disorder, while Lee and colleague (Lee et al., 2025a) reported that VR-based cognitive behavior therapy alleviated suicidality in depressed patients. However, their samples included more severely ill patients. Our study complements this work by focusing on a younger population with mild-to-moderate depression. Barsasella et al. (2021) found that VR interventions improved quality of life and functional fitness in older adults, but their focus on physical outcomes differs from our emphasis on emotional and sleep-related outcomes.

Lee et al. (2024) constructed a standalone immersive meditation intervention; all virtual scenarios were tranquil static landscapes designed for sustained mindfulness practice, with structured mindfulness guidance running through the whole VR session to target long-standing depressive trait symptoms. (Lee et al., 2025a).further integrated systematic cognitive behavioral therapy (CBT modules) into virtual environments, embedding cognitive restructuring, automatic thought correction and anhedonia intervention links within the VR workflow. The two sets of interventions were purpose-built to tackle persistent, trait-based depressive manifestations measured by HAMD-17 and SAS, including feelings of worthlessness, sustained low mood and chronic somatic anxiety, which is why their trials detected broad improvements on clinical depression rating scales. Barsasella et al. (2021) carried out VR intervention targeting elderly participants, with the core design centered on low-intensity fitness activities. Their main observation indicators were physical activity levels and life satisfaction, rather than affective and depressive psychological scales. Their research focused on physical functional improvement of the elderly, which cannot be directly extended to young college students troubled by frequent daily negative rumination and sleep disturbance.

By contrast, the IVR protocol adopted in the present study takes action-adventure gameplay as the primary intervention medium. Participants completed a series of embodied physical interactions such as climbing and exploring in virtual open worlds for 30 min, followed by only a brief 10-min guided relaxation segment without standardized cognitive restructuring training. This game-centered immersive design creates strong real-time diversion from daily repetitive rumination, which generates immediate and cumulative relief of transient, situational diffuse negative mood—this perfectly accounts for the prominent dose–response effect we observed on the PANAS negative affect subscale across all follow-up time points. However, without targeted cognitive correction modules to reshape stable depressive cognitive schemas, the game-based IVR scheme cannot produce measurable reductions in trait-level core depressive and anxiety symptoms, resulting in non-significant intergroup differences on HAMD-17 and SAS after the 12-week intervention.

This content-driven discrepancy between existing meditation/CBT VR and the action-game IVR model adopted here offers a clear interpretative framework for our mixed statistical results. Game-style immersive VR exerts prominent regulating effects on short-lived, reactive negative affective states unique to student daily life, while CBT-embedded demonstrates greater potential for ameliorating persistent clinical depressive symptoms. For future research, hybrid IVR protocols combining adventure gameplay for mood distraction and built-in CBT cognitive modules can be developed, to simultaneously relieve students’ daily negative mood and improve core depressive clinical manifestations.

### Strengths and limitations

This study has several notable strengths. First, as a pilot RCT with rigorous randomization, blinding of intervention providers, and separate living/learning spaces for the two groups, it minimizes selection bias and intergroup contamination, enhancing internal validity. Second, the use of multiple validated psychological assessment tools allows for a comprehensive evaluation of intervention effects across multiple mental health dimensions. Third, the structured IVR intervention protocol—utilizing the Sony PSVR2 device and the Horizon: Call of the Mountain game with a standardized 40-min session design—ensures consistency and reproducibility. Finally, the focus on college students, a high-risk population for depression with unique psychological and developmental need (Sun et al., 2024), addresses an unmet clinical need and provides tailored evidence for this group.

A major methodological limitation of this pilot trial lies in the absence of an active matched control group, which prevents us from disentangling the discrete therapeutic contributions of multiple integrated intervention components. The experimental IVR arm combined immersive visual stimulation, interactive physical gameplay, structured evening relaxation and mindfulness training, as well as extended weekly face-to-face contact time with researchers. In contrast, the control group only received basic routine campus psychological guidance without matched activity or equal contact frequency. Therefore, we cannot separately quantify whether the significant improvements in negative affect originate from immersive VR exposure, physical movement, game-based distraction, relaxation practice, extra clinical interaction, or general placebo/expectation effects. As this feasibility study only aimed to explore the overall auxiliary value of the complete IVR intervention package for depressed college students, dissecting individual component effects was not within the scope of the current research objectives. Subsequent large-scale confirmatory trials will adopt a multi-arm design with several active control conditions (non-immersive screen games, offline mindfulness sessions with identical contact duration) to isolate the unique effects of each intervention module and rule out expectation bias.

Consistent with classical affective psychology theories, emotion refers to short-lived, discrete psychological responses triggered by specific stimuli. Anhedonia and loss of interest are typical manifestations of such responses and represent core clinical symptoms of depression. In contrast, affect stands for persistent, diffuse background mood with low situational specificity, which corresponds to the psychological construct measured by the Negative Affect subscale of the Positive and Negative Affect Schedule (PANAS-NA). Although PANAS was used as an assessment indicator for college students with depression in this study, potential limitations and measurement biases may exist. Follow-up studies should prolong intervention cycles and recruit student samples from multiple regions to enrich existing evidence, so that PANAS can be widely adopted as a universal measurement tool for depression among college students in future research.

All psychological outcome results reported herein are only preliminary exploratory observations and cannot support definitive causal inferences about IVR therapeutic effects. Future large-scale clinical trials will conduct precise sample size calculations based on the effect sizes derived from this study to ensure sufficient statistical power for between-group comparisons. Finally, we plan to carry out a formal large-sample RCT in follow-up research equipped with a long-term follow-up mechanism. Regular online reminders and flexible assessment time slots will be implemented to track the sustained effects of the intervention over an extended period and accumulate more adequate research data.

### Implications for practice and future research

The findings of this study have important implications for clinical practice in college mental health settings. Given the high prevalence of depression among Chinese college students (24.1%) (Yuan et al., 2024) and the limitations of conventional treatments (e.g., low adherence, side effects, limited efficacy) (Khalifeh et al., 2023; Montano et al., 2023), IVR offers a promising adjunctive intervention. Its non-pharmacological nature, engaging format, and targeted efficacy in reducing negative emotions and improving sleep make it particularly appealing to young adults, who may be more receptive to technology-based intervention (de Zambotti et al., 2022). Mental health practitioners in university counseling centers may adopt this IVR scheme as an auxiliary tool targeted at relieving persistent negative mood for students with mild-to-moderate depressive symptoms. Importantly, this intervention should not be regarded as a formal clinical treatment for depressive disorder given the absence of significant improvements on HAMD-17. Future definitive randomized controlled trials should perform formal sample size calculations based on the NA and PSQI effect sizes observed in this pilot study to ensure sufficient statistical power. Moreover, future trials will adopt multi-arm parallel designs with matched active control groups to disentangle the independent therapeutic contributions of immersive VR, physical activity, gamification and mindfulness training, and eliminate confounding effects brought by extra contact time and participant expectation/placebo responses.

## Conclusion

In conclusion, this pilot randomized controlled trial demonstrates that standardized immersive virtual reality (IVR) functions as a targeted adjunctive tool for mitigating negative affect in college students presenting mild-to-moderate depressive symptoms. Notably, no statistically significant intergroup reductions in clinical depression severity (HAMD-17), anxiety or perceived stress were observed following the IVR program, and only a non-significant trend of sleep improvement was detected. The intervention produces specific benefits for transient negative affective distress unique to student populations but cannot be regarded as a broad treatment for clinical depressive disorder. Its accessible, game-based design renders it a useful supplementary resource for campus mental health services focusing on emotional regulation. Subsequent large-scale definitive trials are needed to replicate the observed negative affect benefits and explore whether optimized IVR protocols can generate measurable improvements on core clinical depression rating scales before drawing broader clinical treatment inferences.

## Data Availability

The original contributions presented in the study are included in the article/supplementary material, further inquiries can be directed to the corresponding author.
